# Using averaged models from 4D ultrasound strain imaging allows to significantly differentiate local wall strains in calcified regions of abdominal aortic aneurysms

**DOI:** 10.1007/s10237-023-01738-x

**Published:** 2023-07-05

**Authors:** Achim Hegner, Andreas Wittek, Wojciech Derwich, Armin Huß, Antonio J. Gámez, Christopher Blase

**Affiliations:** 1 Personalized Biomedical Engineering Lab, Frankfurt University of Applied Sciences, Frankfurt am Main, Germany; 2 Department of Vascular and Endovascular Surgery, Goethe University Hospital, Frankfurt am Main, Germany; 3 Department of Mechanical Engineering and Industrial Design, School of Engineering, University of Cadiz, Cadiz, Spain; 4 Cell and Vascular Mechanics, Goethe University, Frankfurt am Main, Germany

**Keywords:** In vivo, Local strain, Abdominal aortic aneurysm, 4D ultrasound strain imaging

## Abstract

Abdominal aortic aneurysms are a degenerative disease of the aorta associated with high mortality. To date, in vivo information to characterize the individual elastic properties of the aneurysm wall in terms of rupture risk is lacking. We have used time-resolved 3D ultrasound strain imaging to calculate spatially resolved in-plane strain distributions characterized by mean and local maximum strains, as well as indices of local variations in strains. Likewise, we here present a method to generate averaged models from multiple segmentations. Strains were then calculated for single segmentations and averaged models. After registration with aneurysm geometries based on CT-A imaging, local strains were divided into two groups with and without calcifications and compared. Geometry comparison from both imaging modalities showed good agreement with a root mean squared error of 1.22 ± 0.15 mm and Hausdorff Distance of 5.45 ± 1.56 mm (mean ± sd, respectively). Using averaged models, circumferential strains in areas with calcifications were 23.2 ± 11.7% (mean ± sd) smaller and significantly distinguishable at the 5% level from areas without calcifications. For single segmentations, this was possible only in 50% of cases. The areas without calcifications showed greater heterogeneity, larger maximum strains, and smaller strain ratios when computed by use of the averaged models. Using these averaged models, reliable conclusions can be made about the local elastic properties of individual aneurysm (and long-term observations of their change), rather than just group comparisons. This is an important prerequisite for clinical application and provides qualitatively new information about the change of an abdominal aortic aneurysm in the course of disease progression compared to the diameter criterion.

## Introduction

An Abdominal aortic aneurysm (AAA) is a degenerative disease of the aorta characterized by a permanent local dilatation. The major hazard is rupture of the aneurysm wall (Frömke 2006) which is associated with a mortality of approximately 52% (Torsello et al. 2016; Kühnl et al. 2017). Clinical indications for surgical treatment are a maximum diameter of the AAA (>50 mm for women and >55 mm for men) and a growth rate greater than 10 mm/year (Wanhainen et al. 2019). But the current criterion provides only a rough estimate of rupture risk because adverse events may occur in aneurysms that do not meet the surgical criteria (Nicholls et al. 1998), whereas large aneurysms may remain stable throughout life (Darling et al. 1977; Farotto et al. 2018). Thus, it is generally accepted that additional patient-specific decision criteria and/or new patient-specific information to characterize the elastic properties of the AAA vessel wall are needed. (Vorp 2007; Humphrey and Holzapfel 2012; Kontopodis et al. 2015). In the healthy state, the aortic wall can be described as an anisotropic, nonlinear elastic, and multilayered fiber-reinforced composite (Fung 1993; Humphrey 2002; Nichols and McDonald 2011). A multifactorial process, that promotes pathologic remodeling like elastin degradation, leads to a weakening and enlargement of the vessel wall (Niestrawska et al. 2019). The mechanisms responsible for these changes are still not fully understood (Farotto et al. 2018). In addition, an intraluminal thrombus (ILT) and calcified wall regions are observed in many AAAs which may cause further complications. Calcifications are solid calcium deposits that are embedded in the soft matrix of the vessel wall and affect its integrity. Local stiffness is known to be significantly increased by calcifications (Volokh and Aboudi 2016) and associated with increased risk of rupture (Buijs et al. 2013; O’Leary et al. 2015). However, the exact influence of calcifications on biomechanics is also not yet fully understood (Farotto et al. 2018).

All of the mentioned changes lead to increased heterogeneity of wall properties and weakening of its structural integrity promoting mechanical failure (rupture). Rupture is a local event which requires local assessment of the changes in wall properties. Global parameters give only a rough indication and often fail to assess individual rupture risk (Gasser 2016). The description of local strain as well as distensibility distributions have emerged as additional potential biomarkers for the assessment of local rupture risk. A decrease in distensibility over time is associated with a significantly reduced time to rupture (independent of age, gender, max. AAA diameter or diastolic blood pressure) (Wilson et al. 2003; Hoegh and Lindholt 2009) and therefore would be a suitable biomarker for predicting rupture risk. Similarly, in addition to different strain limits, different regions of an AAA show severe overstretching, particularly in the surrounding regions of calcification (Thubrikar et al. 2001; Barrett et al. 2018). These changes in local elastic properties are of particular interest because thoracic aneurysms are known to rupture not always in the area of highest stress but in weakened zones of the tissue in which strain localizations and local thinning of the wall can be found (Romo et al. 2014). Both conditions are caused by a damage to the arterial wall, and similar risk factors such as hypertension, smoking, and genetic factors can promote the development of aneurysms in both regions (Golledge 2019), which may be an indication of related rupture behavior.

In recent years, time-resolved 3D ultrasound strain imaging (4D-US) has been increasingly used to provide volumetric assessment of geometry and rupture risk of AAAs (Rouet et al. 2010; Long et al. 2013; Kok et al. 2015) and risk assessment based on the wall’s mechanical properties (Gasser et al. 2010; Wittek et al. 2013; van Disseldorp et al. 2016a, 2019; Petterson et al. 2019) noninvasively. However, the results of biomechanical models rely heavily on an accurate description of AAA wall geometry on the one hand (making it one of the most important factors toward the robust prediction of individual AAA material behavior (Gasser et al. 2022)), as well as on the boundary conditions given (among others) by a robust motion tracking and strain mapping on the other hand. 4D US-based measurement and reconstruction of the 3D geometries of the heart and of large blood vessels have been validated in vitro and in vivo (Soliman et al. 2008; Seo et al. 2009; Park et al. 2011; Seo et al. 2011; Bihari et al. 2013; Kok et al. 2015; Alessandrini et al. 2016; Derwich et al. 2016). Limitations in the use of ultrasound to capture vessel geometry are the limited field of view, angular dependence, refraction, shadowing artifacts and low arterial wall contrast and resolution in the lateral direction. Especially the latter limitation affects in vivo imaging and leads to difficult segmentation and strain measurements, which ultimately affects the results of biomechanical models (Petterson et al. 2021a). Methods to improve image quality, measurement accuracy and motion tracking (Foster et al. 1993; Righetti et al. 2003; Shao et al. 2019; van Hal et al. 2023) and multi-view/multi-perspective ultrasound imaging to improve anisotropic contrast and resolution by increasing the signal/contrast-to-noise ratio (Zimmer et al. 2018; Peralta et al. 2018; de Hoop et al. 2020; Petterson et al. 2021b, a) have been introduced. However, these approaches often require specific changes to the data acquisition hardware and / or software which prevents their use in a current clinical setting.

Our group uses 4D-US strain imaging to measure the deformations of AAAs in vivo. Based on these imaging data, the in-plane strain tensors could be calculated and in turn, the calculation of statistical distribution indices (DIs) such as mean and peak strain, heterogeneity index, and local strain ratio could be proposed to quantify differences between aortic and aneurysm wall motion. These new mechanical biomarkers capture the spatial heterogeneity of individual wall motion and local elastic properties in the physiological range for clinical purposes (Karatolios et al. 2013; Wittek et al. 2017, 2018; Derwich et al. 2016, 2020, 2021).

In an in vitro validation study performed by our group (Wittek 2020), the uncertainty and reproducibility of a single 4D US measurement was quantified, as common in clinical practice. Using an inflation-extension device, a tubular specimen of porcine aorta was loaded physiologically by axial pre-stretch and cyclic pressure change. Cyclic deformations/strains were measured in parallel by 4D-US and optically using an orthogonal two camera setup. The measurement accuracy in the lateral direction showed no significant difference to the beam direction. Although no systematic error in repeated measurements of different subjects was observed, a considerable random error (IQR > 1% for circumferential strain) was identified. This may result in a relative error of $\ge$ 100% in AAAs where mean strains between 0.5 and 3.0% are observed, usually (Batagini et al. 2016; Wittek 2020; Li et al. 2021; Derwich et al. 2021). Global group differences between young, older atherosclerotic, and aneurysmal aortas were successfully determined (Derwich et al. 2016; Wittek et al. 2018). But single measurements, like done in clinical practice, cannot be used for reliable assessment of individual cases/patients or results of biomechanical models. They are valid for group comparisons only, where the random error is averaged out by the number of included patients.

This study aims to develop a method to combine multiple motion functions based on one ultrasound measurement into an averaged function, since averaging is a suitable way to minimize statistical noise. This should reduce the random error of local strain measurements and allow discrimination of individual patients and not only patient groups. Furthermore, the influence of different tissue composition on the deformation behavior of AAAs is investigated using single 4D-US measurements and averaged models. For this, calculated local strains are then divided into areas with and without calcifications, because the rigid structure of these should result in significantly smaller strains in these areas. Subsequently, it is investigated whether significant differences can be found among individual patients based on a single ultrasound measurement and averaged models. This is a basic requirement for future clinical applications and also the validity of biomechanical models.

## Methods

**Fig. 1:**
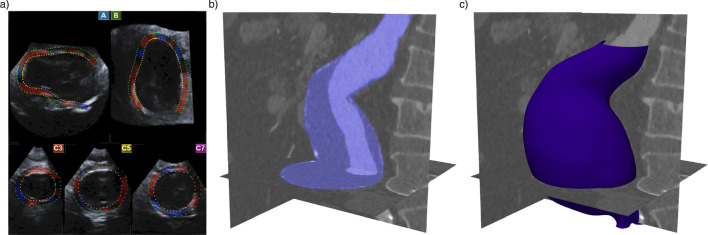
AAA of patient 3 with a maximum diameter of 64.0 mm. **a** Shows five 2D cross section of a volumetric ultrasound data set, two longitudinal cross sections in the sagittal (A) and frontal (B) plane and three transversal cross sections (C3, C5, and C7) at different positions along the AAA. Segmentation of the vessel wall area as region of interest for the wall motion tracking was performed manually in the A and B planes and then automatically completed by the algorithm in the whole image volume. The false masking inside the vessel is called pseudo-apex and will be removed in the further model setup. **b** The same AAA as in image a) based on CT-A imaging. The complete AAA is highlighted. The darker areas are the ILT, the light area is the blood perfused lumen with contrast agent, and the bright shining areas around the vessel wall are calcifications. **c** Same image as in b) with the reconstructed vessel wall as surface plot after smoothing

### Overview and data structure

An overview of the data is given here first, before the details are discussed in the following sections.

For each patient:

1. Computed tomography angiography (CT-A) and 4D ultrasound imaging (4D-US) of the AAA was done (Ch. 2.2).
2. In the CT-A images, aneurysm wall and the calcifications were segmented (Ch. 2.3.1 and Ch. 2.3.2).
3. In the 4D-US images, aneurysm wall was segmented ten times in the end-diastolic configuration $f^0$ (Ch. 2.3.3), followed by a speckle tracking (Ch. 2.4). Each segmentation thus contains an estimate of the AAA motion function for one cardiac cycle.
4. An averaged model was built from these ten segmentations of the same ultrasound data set (Ch. 2.5 and Appendix A).
5. For the ten segmentations as well as for the averaged model, the in-plane strain tensor was calculated in local coordinates with end-diastole as reference configuration $f^0$ (Ch. 2.6 and Appendix B).
6. The US geometries were registered to the CT-A geometries (Ch. 2.8).
7. After manual subdivision of strains into areas with and without calcification, distribution indices (DIs) were calculated and statistically compared (Ch. 2.7, 2.8, 2.9 and 2.10).

The following chapters describe the individual steps in detail.

### Study group and data acquisition

Data of ten AAA patients (eight male, two female) with age 74 ± 8 years (mean ± sd) presented at the Clinics for Vascular and Endovascular Surgery of the University Hospital Frankfurt am Main were evaluated for this study. The study was approved by the local ethics committee. An abdominal aorta was defined as aneurysmal when its maximum diameter exceeded 30 mm. Maximum diameter of included AAAs as determined by clinical measurement using 2D ultrasound was 49.6 ± 8.3 mm (mean ± sd). 4D Ultrasound imaging of the AAAs and Computed Tomography Angiography (CT-A) for the abdomen were performed for each patient. Ultrasound imaging was done at the University Hospital Frankfurt am Main in Germany by the same experienced observer. Examination of patients was carried out in supine position after 5 min of rest. 4D ultrasound data were acquired by use of a commercial real-time 3D-echocardiography system (Artida, Toshiba Medical Systems, Otawara, Japan) that was equipped with a 3D transthoracic probe (Toshiba, PST-25SX, 1-4 MHz phased array matrix transducer). The measurement was triggered by ECG with end-diastole as starting point (reference) of each cardiac cycle. In each case six subvolumes of 90$^\circ \times$15$^\circ$ were recorded over six consecutive heart cycles and merged by embedded Toshiba software resulting in a ECG triggered full volume data set of 90$^\circ \times$90$^\circ$. Wavelength of the ultrasound signal was 0.39 mm at a frequency of 4 MHz, frame rate was 27.1 ± 4.9 fps allowing 23 ± 7 different deformation states of the AAAs to be collected over one cardiac cycle at a heart rate of 63 ± 18 bpm and a resolution of 0.54 ± 0.10 mm/voxel (mean ± sd, $n = 10$, respectively). Field of view between patients differs because of variations in the depth position of the aneurysm (mainly due to increased BMI). The length and width of the field of view vary between 8.8 and 12.9 cm at 6.1$-$11.8 cm depth.

Except for the patients 5 and 7 CT-A imaging was also performed at the University Hospital Frankfurt am Main. Mean slice thickness of the CT-A images was 1.53 ± 0.6 mm.

Patients were included if image quality showed no artifacts, wall and surrounding tissue contrast allowed successful segmentation and post-processing (speckle tracking) using the Toshiba ACP package, there was a CT-A scan in addition to the ultrasound measurement, and a maximum of 8 weeks elapsed between US and CT-A imaging (mean duration between CT-A and ultrasound imaging were 17 ± 22 days).

### Segmentation of patient specific geometries

**Fig. 2:**
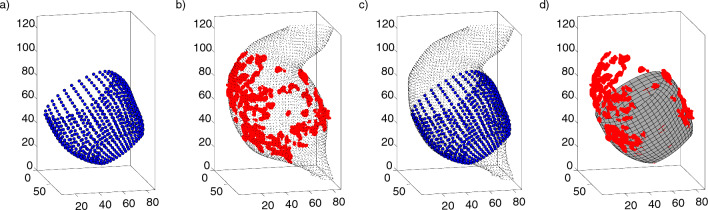
Comparison of ultrasound and CT-A AAA geometries for patient 3. **a** Ultrasound wall geometry after removing of the pseudo-apex. **b** CT-A geometry showing the vessel wall (small points, black) and calcification (big points, red). **c** Ultrasound geometry (big points, blue) and CT-A geometry (small points, black) after registration (cf. Ch. 2.8) **d** Calcification segmented from CT-A images (red points) and ultrasound geometry represented as 2D elements after registration (rectangle mesh, gray). The dimensions of all three coordinate axes are given in [mm], resolution of wall and calcification from CT-A imaging are down-sampled for better presentation

#### Segmentation of the vessel wall from CT-A images

The Vascular Modeling Tool Kit (VMTK, version 1.4.0, www.vmtk.org) was used to reconstruct the blood vessel walls. All AAAs showed a moderate to severe thrombus around the lumen. Because of the very low difference in contrast between the thrombus and the surrounding tissue, no automated segmentation method was used. The vessel wall was segmented piece by piece using the Colliding Fronts method with manual input of the local Hounsfield units (HU). This results in a fine demarcation of the vessel wall and a resulting geometry which consists of approx. 60,000 points. After segmentation, the extracted vessel structure was converted into a surface using the Marching Cubes algorithm (Lorensen and Cline 1987). The resulting surfaces were smoothed using the Taubin algorithm, which keeps the geometry from shrinking during smoothing (Taubin 1995) (see Fig. 2b, c).

**Fig. 3:**
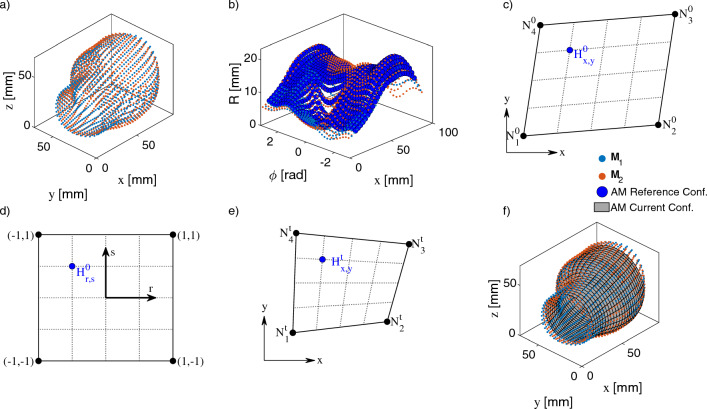
**a** Ultrasound-based AAA geometries of patient 1 from two different segmentations ${\varvec{M}}_1$ and ${\varvec{M}}_2$ in global Cartesian *x*-*y*-*z* coordinates in the reference configuration $f^0$. **b)** Unwinding of segmentations ${\varvec{M}}_1$ and ${\varvec{M}}_2$ of the AAA geometries from image a) in cylindrical coordinates (small dots). The large dots show the homogeneous re-mesh of segmentation ${\varvec{M}}_1$ based on interpolation function in the *x*-$\phi$ and *R*-$\phi$ planes for equally distributed coordinates in the reference configuration $f^0$. **c** An arbitrary element (connected points) of the segmentation ${\varvec{M}}_1$ with vertices $N_1^0$-$N_4^0$ in the reference configuration $f^0$ in global Cartesian coordinates. Inside the element the assigned point of the homogeneous mesh $H_{x,y}^0$ with coordinates $x_H$ and $y_H$. **d** Isoparametric geometry transformation of both element and homogeneous point from image c) into a local *r*-*s* coordinate system located at the centroid of the element for the reference configuration $f^0$. The homogeneous point *H* here has coordinates $r_H$ and $s_H$ in the limits -1:1. **e** The same element from image (**c**) and (**d**) in the following due to the blood pressure distorted current configuration $f^t$ with vertices $N_1^t$-$N_4^t$ (continuous lines). The homogeneous point $H_{x,y}^0$ from image (**c**) created in the reference configuration $f^0$ moves through the linear shape functions proportionally based on the ultrasound measured deformation of the element to position $H_{x,y}^t$ in the current configuration $f^t$. **f** The two initial segmentations ${\varvec{M}}_1$ and ${\varvec{M}}_2$ from figure (**a**) and the averaged model created from these two segmentations with a homogeneous mesh after back transformation into Cartesian coordinates for the current configuration $f^t$

#### Segmentation of calcifications from CT-A images

Software tools for automated determination of calcification from CT-A images are generally designed to measure coronary artery calcification or have been validated only for this purpose. There is no evidence for the reliability and accuracy of these methods for measuring abdominal aortic calcification because differences in coronary and abdominal CT acquisition may influence the results of these tools (Buijs et al. 2018). Additionally, the presence of intravascular contrast agent has a serious impact, as it results in significant volume and mass differences (Buijs et al. 2018). Likewise, small calcifications are prone to mismeasurement, as one voxel of a CT scan may contain multiple tissue regions, but only one HU value is determined by averaging. Automated tools most commonly operate with a threshold of 130 HU, which has been used in studies in coronary artery calcification (Agatston et al. 1990; Budoff et al. 2007; Carr et al. 2017), thoracic aortic calcification (Cury et al. 2004; Allison et al. 2009), and abdominal aortic calcification (Davila et al. 2006; Allison et al. 2009). In this study, we used a region growing algorithm in VMTK, which has the advantage that HU levels can be set manually for segmentation to avoid volume and mass differences, instead of tools which use a fixed HU value. In patients for whom CT-A images without contrast agent were available, the established threshold of 130 HU was chosen for reconstruction of the calcifications. For the two patients 9 and 10, for whom only CT-A images with contrast medium were available, the threshold was increased to 210 HU and 235 HU on a patient-specific basis, as suggested by Buijs et al. (2018) (see Fig. 2b, c, d).

#### Segmentation of the vessel wall from 4D ultrasound images

Post-processing of the 4D ultrasound data was performed using the commercial Advanced Cardiac Package (ACP, Toshiba Medical Systems, Otawara, Japan). Spatial position of the tissue region was determined from the measured time of flight on the assumption of a constant speed of sound of 1540 m/s, which holds well for tissues in the human abdomen (Goss et al. 1980). Transition between aortic wall and lumen could be identified clearly due to the difference in acoustic impedance between tissue and blood. However, this difference does not hold between wall and surrounding tissue, why wall thickness cannot be measured. Tissue close to the luminal border of the aortic wall as region of interest for later motion tracking was segmented manually in two longitudinal cross sections of the volumetric image (A and B Fig. 1a). Based on this segmentation, the software completed the three-dimensional region of interest automatically. Three additional transversal cross sections of the volumetric data set (C3, C5 and C7 planes in Fig. 1a) allowed the observer to control the automatic completion of the segmentation.

### 3D wall motion tracking

In the US images, the aneurysm wall was segmented ten times each for every of the ten patients by an experienced observer. Thus, one ultrasound segmentation consists of a manual geometry segmentation in the end-diastolic configuration and a subsequent estimation of the motion function of the AAA based on the speckle tracking algorithm. For each of these ten segmentations, several different deformation states result for one cardiac cycle. Since they are from the same ultrasound image, the number of deformation states in these ten segmentations is identical, but can be different for each patient. In terms of continuum mechanics, the deformations states can be interpreted as configurations *f*. End-diastole can be interpreted as the reference configuration $f^0$, while the following deformation states can be interpreted as current configurations $f^t$. Thus, one configuration (reference $f^0$ or current $f^t$) of one of these ten segmentations represents one of ten deformation estimates of the identical time point within the cardiac cycle.

The speckle tracking algorithm provided by the commercial ACP used cubic template volumes of approximately 10$\times$10$\times$10 $\text {mm}^3$ for pattern recognition by means of 3D-correlation in subsequent time steps (Chen et al. 2005). The centroid of these template volumes was used for motion estimation (Seo et al. 2009, 2011) for each time step throughout the cardiac cycle. Fields of 1296 position vectors of motion estimation points in Cartesian coordinates were exported. After removal of the artificial pseudo-apex (see Fig. 1a), fields of between 504 and 828 motion estimation points remained. These can be understood as discrete material points *X* in a continuum mechanical sense (Ogden 1997; Holzapfel 2010) that describe different deformed configurations (here referred to as current configurations $f^t$) of the aortic and aneurysmal wall throughout the cardiac cycle. The material points $X_{i, j}$ defining the aortic wall geometry were arranged into subsets of 36 points for different ‘heights’ along the longitudinal axis of the aortic segments. $X_{i, j}$ is the $j^{th}$ discrete material point within the $i^{th}$ ‘height’ or subset along the length of the imaged segment, $j = 1,...\,,36$ and $i = 1,...\,,n,\, n<36$ (Wittek et al. 2018) (see Fig. 2a,c, d). Resulting lengths of the blood vessels after trimming the pseudo-apex where approximately 38.8$-$64.1 mm.

In the following chapters, the first segmentation is referred to as ${\varvec{M}}_1$, the reference configuration of the first segmentation is referred to as ${\varvec{M}}_1^0$ and a current configuration of the first segmentation is referred to as ${\varvec{M}}_1^t$. For the other segmentations, the lower index is adjusted.

### Creation of averaged models

Averaging multiple segmentations can be done by combining the closest points of each segmentation into one point. However, this often results in a highly distorted mesh and has a negative effect on subsequent calculations. Therefore, we propose a more elaborate method here, which guarantees a homogeneous mesh. The method works with any number of segmentations, which can contain different numbers of nodes. For a better overview, the example shown uses only two segmentations. The method can be found with detailed formulations and mathematical descriptions in Appendix A, only a brief description is given here, to condense the main part of the paper.

Let us consider two segmentations ${\varvec{M}}_1$ and ${\varvec{M}}_2$ from Fig. 3a. The geometries of the reference frame $f^0$ of both segmentations are transformed into polar coordinates and then interpolated (see Fig. 3b) to create new points $H_{x,y}^0$ at identical positions in both segmentations, which then form a homogeneous mesh. Each of these new points is then assigned to an original element measured by ultrasound (see Fig. 3c, $N_{1}^0$-$N_{4}^0$). The basic idea of the method is to maintain this relative position of a point $H_{x,y}^0$ in the assigned element over the cardiac cycle. For this purpose linear shape functions according to Eq. 12 are used. After isoparametric geometry transformation of homogeneous point and associated element (see Fig. 3d), the tracking of the point over one cardiac cycle (configurations $f^t$) is described by the deformation of the element measured by ultrasound (see Fig. 3e, $N_{1}^t$-$N_{4}^t$, Eq. 13). After back-transforming and averaging the respective identical homogeneous points of all segmentations in all configurations $f^t$, an averaged model based on the ultrasound measured element deformations results, which is exactly what we want (see Fig. 3f).

### Calculation of Biot’s in-plane strain tensor

4D ultrasound data define the discrete motion function

1 $$\begin{aligned} {\varvec{x}}_{i, j} = \chi ({\varvec{X}}_{i, j}, t) = {\varvec{X}}_{i, j} + {\varvec{u}}_{i, j}(t),\end{aligned}$$

where ${\varvec{x}}_{i, j}$ is the position vector of a discrete material point at the time *t* of the cardiac cycle, $\chi$ is the motion function, ${\varvec{X}}_{i, j}$ is the position vector of the same material point $X_{i, j}$ in the chosen (deformed) end-diastolic reference configuration, and ${\varvec{u}}_{i, j}$ is the displacement vector which describes the motion from the reference $f^0$ to any imaged current configuration $f^t$.

Calculation of Biot’s in-plane strain tensor was implemented in-house in MATLAB and verified by the commercial finite element solver Abaqus 6.12 (Simulia - Dassault Systèmes, Vélizy-Villacoublay, France). The end-diastolic position identified by ECG-triggered ultrasound serves as a reference configuration $f^0$, which does not describe an undeformed configuration in blood vessels due to axial and circumferential pre-strain (Horny et al. 2012; Sokolis et al. 2017). The deformation was quantified by the Biot’s strain tensor, which is free of rotations and suitable for large deformations. It was defined as

2 $$\begin{aligned} \varvec{\epsilon } = {\varvec{U}} - {\varvec{I}} \end{aligned}$$

where ${\varvec{I}}$ is the second-order identity tensor and ${\varvec{U}}$ is the right strain tensor. Linear 2D shape functions according to Eqs. (11) and (12) were also used here with integration point at $r=s=0$ (element centroid). The in-plane components of the strain tensor were calculated for each configuration in local element coordinate systems, each localized at the element centroid. Here, the 1-axis corresponds to the longitudinal and the 2-axis to the circumferential direction of the blood vessel. These in turn were recalculated for each deformed configuration to account for solid body motion during the cardiac cycle. A detailed design description of the coordinate systems can be found in Wittek et al. (2016). A detailed mathematical representation of the calculation of the in-plane Biot’s strain tensor $\varvec{\epsilon }$ can be found in Appendix B.

### Distribution indices

To characterize the strain distribution of the aneurysmal wall quantitatively, we introduced simple statistical distribution indices (DIs) (Karatolios et al. 2013; Derwich et al. 2016; Wittek et al. 2018):

*Mean and max strain*: The arithmetical mean and maximum of the strain distribution.

*Local strain ratio*: It was defined as the ratio of local maximum and mean strain.

*Heterogeneity index*: The coefficient of variation, i.e., the ratio of the standard deviation and the mean of the strain distribution.

To obtain one strain value for each element describing the whole cardiac cycle, DIs were calculated from strain peak-to-peak amplitudes. These strain amplitudes are the difference between the largest and smallest local longitudinal and circumferential strain occurring for each element. This may compare different points in time, but this value reflects the actual locally occurring strain or elastic behavior of the vessel wall over one cardiac cycle, in which we are primarily interested in this study.

**Fig. 4:**
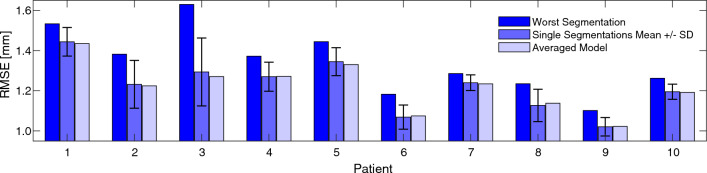
RMSE values for the geometry comparison between ultrasound and CT-A based on a rigid body registration. The left column is the worst segmentation, in the middle the mean value of all ten segmentations and the standard deviation as error bars, and on the right the averaged model, respectively

### Registration of ultrasound and CT-A geometry

Since both geometries are from different imaging modalities, registration of blood vessel geometries must be performed for assignment. For geometry comparison of the segmented blood vessels from both imaging modalities, a rigid body registration is performed, which preserves the shape. For subsequent mapping of calcified areas, an affine registration is applied, which changes the (global) shape until the best possible match is found. To do so, an in-house extended variant of the Iterative Closest Point (ICP) algorithm, originally presented by Besl and McKay (1992) as well as Chen and Medioni (1992), was implemented in MATLAB R2021b. In performing the algorithm, an attempt was made to make a moving point cloud (US) coincide with a static point cloud (CT-A) as well as possible. To find the needed transformation $\varvec{\tau }$ and translation ${\varvec{T}}_t$, the algorithm iteratively performs the four steps: 1. Determination of the closest points of both clouds 2. Calculate the sum of the distance squares of these pairs 3. Estimation of the necessary transformation $\varvec{\tau }$ and translation ${\varvec{T}}_t$ 4. Applying the found transformation $\varvec{\tau }$ and translation ${\varvec{T}}_t$.

In case of a rigid body registration the whole transformation is described by a translation vector

3 $$\begin{aligned} {\varvec{T}}_t = \begin{bmatrix} t_x&t_y&t_z \end{bmatrix}^T \end{aligned}$$

as well as a rotation matrix

4 $$\begin{aligned} {\varvec{M}}_r = {\varvec{R}}_x(\alpha ) \cdot {\varvec{R}}_y(\beta ) \cdot {\varvec{R}}_z(\gamma ), \end{aligned}$$

where $t_x$, $t_y$ and $t_z$ are translational motions in *x-*, *y*- and *z*-direction as well as ${\varvec{R}}_x(\alpha )$, ${\varvec{R}}_y(\beta )$ and ${\varvec{R}}_z(\gamma )$ are the 3D rotation matrices around the *x*, *y* and *z* axes for the angles $\alpha$, $\beta$ and $\gamma$, respectively (Goldstein 1981).

In case of affine transformation scaling matrix ${\varvec{M}}_s$ and shear matrix ${\varvec{M}}_h$ must be added by

5 $$\begin{aligned} {\varvec{M}}_s= & {} \begin{bmatrix} s_x &{} 0 &{} 0 \\ 0 &{} s_y &{} 0 \\ 0 &{} 0 &{} s_z \end{bmatrix} \end{aligned}$$

6 $$\begin{aligned} {\varvec{M}}_h= & {} \begin{bmatrix} 1 &{} h_{xy} &{} h_{xz} \\ h_{yx} &{} 1 &{}h_{yz} \\ h_{zx} &{} h_{zy} &{} 1 \end{bmatrix}, \end{aligned}$$

where $s_x$, $s_y$ and $s_z$ are scale factors for the *x*, *y* and *z* directions and $h_{xy}$ - $h_{zy}$ are shear factors in the given plane. By means of these matrices the rigid body transformation $\varvec{\tau }_R$ and the affine body transformation $\varvec{\tau }_A$ in 3D space are feasible by

7 $$\begin{aligned} \varvec{\tau }_R =\,&{\varvec{M}}_r \end{aligned}$$

8 $$\begin{aligned} \varvec{\tau }_A =\,&{\varvec{M}}_s \cdot {\varvec{M}}_r \cdot {\varvec{M}}_h . \end{aligned}$$

In order to coincide the moving ultrasound point cloud ${\varvec{P}}_{US}$, containing $n\times 3$ points (3 represents the *x*-, *y*- and *z*-coordinates), and the static CT-A point cloud ${\varvec{P}}_{CT}$, containing $m\times 3$ points, in the best possible way, the algorithm minimizes the error functions

9 $$\begin{aligned} \varvec{\chi }_{\text {Rigid}}^2 =&\sum _{i=1}^{n} \left\| (\varvec{\tau }_R {\varvec{P}}_{i\,US} + {\varvec{T}}_t) - {\varvec{P}}_{i\,CT} \right\| ^2 \Rightarrow \text {min} \end{aligned}$$

10 $$\begin{aligned} \varvec{\chi }_{\text {Affine}}^2 =&\sum _{i=1}^{n} \left\| (\varvec{\tau }_A {\varvec{P}}_{i\,US} + {\varvec{T}}_t) - {\varvec{P}}_{i\,CT} \right\| ^2 \Rightarrow \text {min} \end{aligned}$$

until $\varvec{\chi }_{\text {Rigid}}^2$ or $\varvec{\chi }_{\text {Affine}}^2$ does not change any more. Because the rough alignment of both geometries is known, parameter space for rotation and translation are limited to avoid unphysiological registration results for nearly symmetric fusiform aneurysms. Therefore, the determination of the new transformation parameters (step 3) was implemented using the nonlinear least-squares fitting function lsqnonlin allowing parameter boundaries. Vessel pre-alignment was performed manually and the centroids of the two vessel geometries were superimposed as initial values for the algorithm. For efficient determination of the closest points (step 1), the MATLAB function knnsearch with selected *k*-d tree procedure was used, as suggested by Zhang (1994). Rigid body registration was used for the subsequent geometry comparison. For the classification of the elements as “calcified element” or “non-calcified element” an affine registration was used.

To quantify the geometric agreement between US and CT-A geometries, the Root Mean Squared Error (RMSE) between the assigned points of both geometries and the Hausdorff Distance, which describes the largest local deviation, were calculated. Because the exact point in the cardiac cycle is not known for the CT-A images, all time steps of the ultrasound geometries were registered to the CT-A geometry and the one with the smallest RMSE was used for further evaluations. In the further course, for each patient the segmentation with the smallest RMSE is called “best segmentation” and the one with the largest RMSE is called “worst segmentation”.

### Assignment of ultrasound elements to calcified areas

The final assignment of which element of the ultrasound geometry is declared as “calcified element” and which as “non-calcified element” was decided manually by eye using the segmented and superimposed ultrasound geometries and calcifications. Assessment was carried out by an engineer specialized in biomechanics working >5 years with this type of data. Ultrasound elements with less than half of the element area covered by calcification were declared as “non-calcified element”. Very small calcifications, which could not cover at least one element, were neglected (mean element edge length 3.33 ± 1.16 mm (mean ± sd)). For each patient, this resulted in two groups with the longitudinal and circumferential strains for areas with calcification and without calcification, which were then statistically evaluated.

### Statistical evaluation

Statistical analysis of group differences was performed with MATLAB R2021b. Using the Kolmogorov–Smirnov test kstest in MATLAB, none of the groups showed a normal distribution. Therefore, a tail left Mann–Whitney U test was performed for all significance tests using the ranksum function. Thereby the null hypothesis was tested, that strains in areas with calcification and in areas without calcification showed equal medians, against the alternative that the median in areas with calcification was less than in areas without calcification.

## Results

**Table 1:** Strain values for the four different Distribution Indices (DIs) based on the worst (W) and best (B) segmentation (Seg.) as well as for the averaged models (A) in areas with calcification (Calc.) and without calcification (No calc.). The results are given for the circumferential and longitudinal strain components as Median [$Q_1$, $Q_3$], where $Q_1$ and $Q_3$ are the first and third quartile, respectively

DIs	Seg.	Circumferential strain $\epsilon _{22}$		Longitudinal strain $\epsilon _{11}$	
		Calc	No Calc	Calc	No Calc
Mean strain	W	3.3 [2.6, 3.9]	3.3 [2.7, 3.8]	3.9 [3.5, 5.2]	4.6 [2.9, 4.9]
[%]	B	2.6 [2.4, 3.2]	3.2 [3.0, 3.9]	3.4 [3.3, 5.1]	3.9 [3.4, 4.6]
	A	2.2 [2.1, 2.9]	2.9 [2.6, 3.5]	3.0 [2.7, 3.6]	3.4 [2.5, 3.9]
Maximum	W	8.1 [6.4, 10.2]	10.1 [7.6, 11.9]	8.0 [7.2, 11.3]	13.4 [8.6, 15.3]
strain [%]	B	5.7 [4.4, 8.4]	11.2 [8.0, 13.6]	11.9 [6.8, 12.3]	12.9 [9.7, 15.0]
	A	6.6 [4.7, 5.2]	8.4 [6.8, 9.0]	7.3 [5.7, 7.8]	8.1 [6.1, 11.3]
Local strain	W	2.7 [1.8, 3.1]	3.2 [2.6, 3.6]	2.1 [1.9, 2.7]	2.9 [2.6, 3.9]
ratio [-]	B	2.0 [1.4, 2.5]	2.9 [2.6, 4.0]	2.2 [1.9, 2.7]	2.8 [2.7, 4.1]
	A	2.3 [1.8, 2.9]	2.9 [2.4, 3.2]	2.1 [2.0, 2.4]	2.7 [2.2, 2.8]
Heterogeneity	W	0.5 [0.4, 0.5]	0.5 [0.4, 0.6]	0.4 [0.4, 0.5]	0.5 [0.4, 0.6]
index [-]	B	0.4 [0.2, 0.5]	0.5 [0.5, 0.7]	0.5 [0.4, 0.5]	0.5 [0.5, 0.5]
	A	0.4 [0.3, 0.5]	0.5 [0.4, 0.6]	0.4 [0.4, 0.5]	0.5 [0.4, 0.5]

### Geometry comparison

In Fig. 4 the worst single segmentations for each patient compared to the mean value of all segmentations (each given as mean ± sd) and the averaged models are shown. The mean RMSE of the ten segmentations of all patients with 1.22 ± 0.15 mm compared to the mean RMSE of all averaged models with 1.22 ± 0.11 mm shows a good agreement between both geometries, but a larger dispersion for the single segmentations. The differences of the RMSE between the best and worst segmentation range from 0.12 to 0.52 mm in the best and worst case with a mean of 0.24 ± 0.13 mm. In the case of the worst negative outlier in patient 3, the RMSE is 1.63 mm compared to 1.11 mm for the best segmentation and 1.29 mm for the averaged model. The mean values of the maximum locally occurring deviations, given by the Hausdorff Distance (HD), are 5.45 ± 1.56 mm for the single segmentations and 4.66 ± 0.81 mm for the averaged models. Overall, there is good agreement between US and CT-A geometries. No correlation of maximum or mean RMSE/HD with AAA diameter was found.

### Comparison of strains in areas with and without calcifications

Only seven of the ten aneurysms showed calcifications, which is why only these were considered in the strain evaluation. Affine registration decreased the mean RMSE to 0.88 ± 0.09 mm between both geometries. The strain distribution indices for longitudinal and circumferential strains based on the worst/best segmentation and averaged models are given in Table 1. Using the worst segmentation, the mean circumferential strains in areas with calcifications are nearly identical to the strains in areas without calcifications. With the best segmentation they are 17% smaller and in the case of the averaged models 23% smaller in areas with calcifications. Also for the strain ratio, the worst segmentations show the smallest strain difference with -14% compared to -31% and -23% for the best segmentation and the averaged models. For the heterogeneity index, it even shows a 9% larger value than in the areas without calcifications compared to -31% and -21% for the worst segmentation and the averaged models. In general, the models based on the best segmentation and the averaged models show similar strain results when compared to each other. This applies to both calcified and non-calcified areas and to the differences between both types of wall areas. In contrast, the models based on the worst segmentation show divergent strain results in most cases. Interquartile range of the areas with and without calcifications shows a smaller dispersion for the averaged model in most cases, while it is nearly similar for the worst and best segmentations.

**Table 2:** Significance levels for differences of circumferential and longitudinal strains in calcified and non-calcified areas for a tail left Mann–Whitney U test

	Circumferential			Longitudinal		
Patient	W	B	A	W	B	A
1	–	**	*	–	***	***
2	–	**	***	–	–	***
3	**	***	***	–	–	*
4	–	–	**	–	***	***
6	–	***	***	–	–	***
9	–	–	***	–	–	***
10	***	***	***	–	–	–

*, ** and *** indicate that differences are significant at the 5%, 1% and 0.1% level. W and B represent the worst and best segmentation while A represents an averaged model

A similar picture emerges for the longitudinal strains. The dispersion is similar for the best and worst segmentation, while it is smaller for the averaged models (cf. Table 1).

Using a tail left Whitney–Mann U test, it can be seen that the circumferential strains based on the averaged models in areas with calcifications can be significantly differentiated from areas without calcifications for all patients. More precisely, strains in areas with calcifications are significantly smaller than in areas without calcifications for all individual patients at the 5% level (86% of patients are significant at the 1% level and 71% of patients at the 0.1% level). In comparison, 71% of patients could be significantly differentiated at the 5% level using the best segmentation and only 29% using the worst segmentation.

For longitudinal strains based on the averaged models, calcified and non-calcified areas could be significantly differentiated at the 5% level by means of the strain values in six out of seven patients (86%), compared with 29% for the best and 0% for the worst segmentations. Significance levels for all patients and segmentations are given in Table 2.

## Discussion

**Fig. 5:**
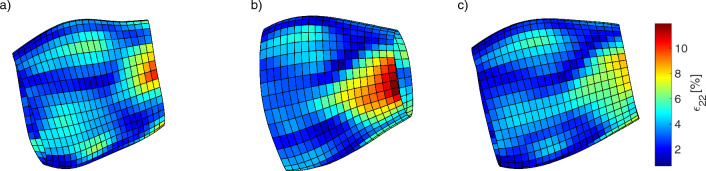
Ultrasound AAA geometries of patient 4 represented as elements based on the (**a**) worst and (**b**) best segmentation as well as the (**c**) averaged model. The colors correspond to the locally occurring circumferential strain amplitudes $\epsilon _{22}$ ranging between 0.70$-$11.91%. The local areas with large strain amplitudes can be most sharply delineated in the best segmentations. Large strain amplitudes are damped by averaging

In this paper, we present a method to create averaged models from multiple ultrasound segmentations and used full-field 4D ultrasound strain imaging to significantly distinguish strains in areas with and without calcifications for individual patients. In an in vitro study carried out by our group (Wittek 2020), no systematic error but a random error (IQR of circumferential strains greater than 1%) was found for repeated US measurements. In single US measurements, however, a systematic error greater than 2% (absolute value) was found. The mean circumferential strain in this study is 1.19 ± 0.51%, which can result in a relative error greater than 100%. Thus, averaging multiple motion functions (cf. Equation (1)) from different segmentations is a suitable method to minimize the random error in order to be able to significantly discriminate individual patients. This was previously only possible for group comparisons (Derwich et al. 2016; Wittek et al. 2018; Derwich et al. 2020).

To test this hypothesis, the in vivo strains of AAAs measured with 4D ultrasound strain imaging were divided into areas with and without calcifications and statistically compared. The respective reconstructions from CT-A images were used as the geometry reference for calcifications and aneurysm wall. CT-A is considered a gold standard for geometry reconstructions mainly because it is a suitable tool for imaging the entire aneurysm and accurately measuring AAA size with high reproducibility (Cho et al. 2020) and has been established in the past as a suitable means for measuring and detecting AAA growth (diameter and volume) with high accuracy and reproducibility (Bargellini et al. 2005; Kauffmann et al. 2011; Parr et al. 2011; Kauffmann et al. 2012) and is thus a suitable reference geometry. Also 3D ultrasound could be found to be a suitable method to determine maximal diameter, vessel area as well as vessel and thrombus volume with excellent agreement compared to CT imaging (Cho et al. 2020). The median Hausdorff Distance found here between the US and CT-A geometries of 5.1 [4.5, 6.1] mm is in good agreement with 4.6 [4.0, 5.9] mm from van Disseldorp et al. (2020), 7.3 [6.5, 10.1] mm from Kok et al. (2015), and 10.8 [9.1, 11.8] mm from van Disseldorp et al. (2016b) (median [$Q_1$, $Q_3$], respectively), who also compared in vivo AAA geometries based on 3D ultrasound measurements with CT reconstructions. Averaged models could achieve a significant reduction in geometry deviation ($p<0.041$) compared to the worst segmentations, which prevents from accidentally evaluating a negative outlier like in the case of patient 3 (see Fig. 4).

A major influence on the results is given by the segmentation of the calcifications and the resulting volume. It has been reported in the past that automated segmentation methods have a sensitivity of 84% and that contrast agent administration leads to an increase in HU limits and thus most likely to deviations. Komen et al. (2011) found that calcification scores with lower HU levels between 130 and 1000 HU did not produce comparable results and more research is needed. We therefore decided to use a semiautomatic segmentation method based on individual HU levels to counteract volume overestimation by averaging the voxels and contrast agent administration (see Ch. 2.3.2). Especially contrast agent administration can lead to HU levels which are higher than in or close to calcified areas. Adequate thresholding and segmentation, as chosen here, using the established 130 HU level for CT without contrast and patient-specific enhancement with contrast, should minimize but not completely prevent overestimation. However, the determination of the “correct” HU level and the influence of volume overestimation on the results of this study cannot be precisely determined, but should be reduced by considering the above measures. Also, in the two patients for whom only contrast CT was available, no significant differences could be found compared to the other patients, which also suggests an increase in HU levels. In summary, the reconstruction of calcifications depends more on postprocessing than on CT-A imaging per se.

However, in addition to geometry comparison, the detection and reliability of local deformations is also necessary. The capabilities of 4D ultrasound to determine local deformations could also be demonstrated by us in a controlled in vitro inflation-extension experiment with porcine aortas under physiological loads (Wittek 2020). The local absolute deviations in circumferential strains there ranged between 0.1 [1.2] and 0.6 [1.1] (given as median [IQR], in %). Seo et al. (2009) have performed in vivo measurements of the left ventricle of ovine hearts. Local reduction of apical wall motion was inducted by occluding the distal left anterior descending coronary artery. Local varying strains were measured by 4D ultrasound speckle tracking and sonomicrometry. Areas with artificially reduced wall motion could be identified clearly and gave evidence of the capability of 4D US speckle tracking to capture local varying and heterogeneous strain fields.

If mean circumferential strain amplitudes are calculated from the difference of global mean maximum and global mean minimal strain (same time point, as calculated in our previous studies), they show mean circumferential strains for all aneurysms of 0.9 [0.6, 1.1] (median, [$Q_1$, $Q_3$] in %). This is in good agreement with our previous findings for AAAs of 0.9 [0.5, 1.2] (median, [$Q_1$, $Q_3$] in %) (Derwich et al. 2016; Wittek et al. 2018; Derwich et al. 2020), and also the findings of the global AAA circumferential strains of 1.88 ± 0.62 (mean ± sd, in %) for large aneurysms reported by (Li et al. 2021) measured using 3D ultrasound, 1.2% reported by (Brekken et al. 2006) and 1.0 [1.0, 2.1] for small and 1.0 [1.0, 1.8] (median, [$Q_1$, $Q_3$] in %, respectively) for large aneurysms reported by (Batagini et al. 2016) using 2D ultrasound. This suggests stability of strain-based measures for in vivo wall characterization. Although the qualitative strain distributions match, averaged models underestimates the maximum strains occurring compared to the single segmentations. Therefore, the difference in the maximum strains and in the strain ratio is larger for the best segmentations than for the averaged models. However, this again underlines the usefulness of the averaged models, since significant differences were found despite attenuation of the maximum strains as shown in Fig. 5. Whether reduced wall thickness in non-calcified areas correlates with high strains cannot be determined here, as ultrasound imaging to date does not provide the ability to determine wall thickness. Also, the volume/thickness of calcifications cannot be measured based on US.

Barrett et al. (2018) performed uniaxial tensile tests on calcified AAA specimens and found mean strain differences of 19% in areas with and without calcifications. This is in good agreement with value of 17% found here for the best segmentation and 23% for the averaged models. The averaged models show a larger difference than the best segmentations. In our opinion, this is due to the fact that the definition of “best” or “worst” masking is based purely on the comparison of both geometries after rigid registration, the strains are not taken into account here. This means that the geometry with the smallest RMSE does not necessarily have the “best strain distributions” or has the strain distribution that corresponds best to the “true” strain distribution, which we do not know. But the smoothing of the strain curves, which results from the averaging of the motion functions, seems to allow a sharper distinction. The main reason for this is that local outliers are smoothed and thus a sharper subdivision is possible, especially in areas with small calcifications. One possible reason for the fact that the values observed in our study are higher is that we used maximum locally occurring peak-to-peak strain amplitudes, the values reported by Barrett et al. (2018) are at a reference strain. Likewise, strain peaks could be found in the marginal areas of the calcifications. These could be a possible explanation for the partly high strains in the calcified areas found in this study. These may have been caused by only slight uncertainties by the registration routine or in the strain fields of the respective segmentation, but which can lead to high strains because of the adjacent strain peaks.

### Limitations and future work

A limitation of this work is that the exact time in the cardiac cycle at which the CT-A scan was taken is not known and blood pressure is not measured during the scan. In addition, there are up to 8 weeks between US and CT-A acquisition, so the relationship between deformation and blood pressure may be different. We therefore made the assumption that the US configuration with the lowest RMSE corresponds to the time of CT-A images taken. Since we used the local maximum element strain from the difference between the maximum and minimum strains to calculate the DIs, this assumption has no influence on the resulting strain curves and does not require knowledge of the time course of the blood pressure curve. However, the greatest influence is on the assignment of the US elements to the calcifications, which is why we used an affine registration. The resulting geometries of all US configurations after affine registration to the CT-A geometry are almost identical, which is mainly due to the low strains in AAAs. Due to the mentioned points, the influence of our assumption should be reduced to a large extent, but cannot be quantified here.

Another point is that averaging from multiple ultrasound images rather than multiple segmentations could reduce the random error even more reliably. However, this was in a controlled in vitro experiment and not in vivo (Wittek 2020), where idealized conditions do not exist. However, if the position of the transducer is changed at each measurement and an averaged model is built from this, there is the advantage that, because of the angular dependence of ultrasound, some areas of the AAA can be better visualized than with constant transducer position. The disadvantage, however, lies in the then necessary registration of the individual recordings using the CT geometry, since they no longer have an identical coordinate basis. It is difficult to assess whether the inaccuracies of the then needed registration negate any benefit of multiple images, since most aneurysms showed a more fusiform and no saccular geometry. This can lead to incorrect alignment of the individual ultrasonic geometries. Therefore, we have chosen to base the analysis on a single ultrasound measurement with multiple segmentations to reduce the random error, which also remains the clinical standard.

However, the method for averaged models presented here also works for multiple images, but requires an uniform coordinate base, which can be achieved (with restrictions) by registration.

In future work, we want to confirm the results on a larger number of patients. The results of this work also offer further, more comprehensive evaluation possibilities using averaged models. We were previously able to significantly distinguish neck and bulge regions of AAAs in a group of 56 patients using DIs (Derwich et al. 2020), which should now also be possible for individual patients. This could also make it possible to identify regions of an AAAs with noticeably altered DIs (calculated based on distensibilities), which could indicate local vulnerabilities or stiffening. Especially in regard of the fact that the stiffness and local wall thickness of AAAs with diameters >50 mm seems to be a better measure of the risk of rupture (Di Martino et al. 2006) and the time to rupture is shortened with increased distensibility (Wilson et al. 2003). The latter evaluated global distensibilities, the use of local distensibilities has the potential to increase the significance even further, but this needs to be investigated in future studies.

### Conclusion

This study demonstrates that averaged motion functions based on in vivo 4D ultrasound strain imaging are capable to significantly differentiate local strains in areas with and without calcifications. Without averaged models, this was previously only possible for group comparisons. In addition, since it is not possible to identify the best segmentation in a conceivable clinical case without a large segmentation effort, the use of averaged models prevents negative outliers. This is an important prerequisite for clinical application and the validity of biomechanical models. The use of significant local information about wall properties has the potential to provide qualitatively new information about the change of an AAA in the course of the disease.

## Figures and Tables

**Fig. 6:**
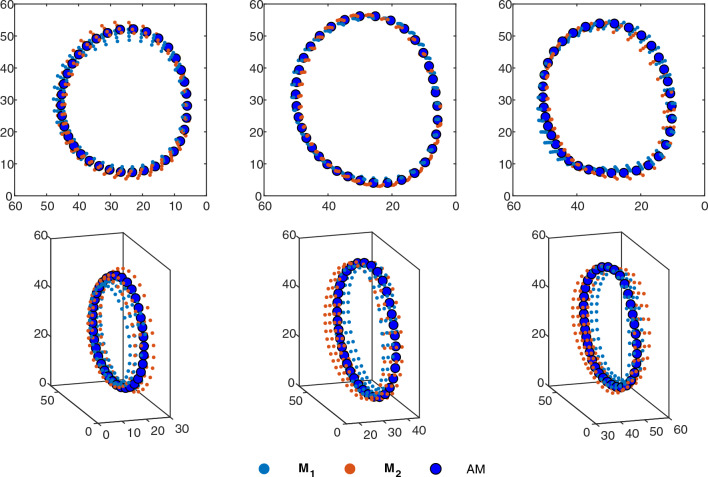
Both segmentation ${\varvec{M}}_1$ and ${\varvec{M}}_2$ as well as the averaged model (AM). Each column shows one of three chosen heights of the averaged model along the longitudinal direction of the blood vessel as well as the assigned elements from both segmentations ${\varvec{M}}_1$ and ${\varvec{M}}_2$ at the systolic configuration $f^{sys}$. The upper row shows the height as 2D slice (*z*-*y*-plane) and the lower row in 3D space. The dimensions of all three coordinate axes are given in [mm]

## Data Availability

Not applicable.

## Appendix A: Mathematical formulation for creating averaged models

The presented method works with any number of segmentations, which can contain different numbers of nodes. For a better overview, the example shown uses only two segmentations.

So let us consider two segmentations ${\varvec{M}}_1$ and ${\varvec{M}}_2$ shown in Fig. 3a, both can have any number of nodes but at least two time steps (a reference configuration $f^0$ and at least one current configuration $f^t$) for one heart cycle. Each node is described in 3D space by an *x*-, *y-* and *z*-coordinate. Since they originate from the same recording, both segmentations already have the same coordinate base.

*Step 1: Remeshing of the reference configuration* $f^0$

In the first step we remesh only the reference configuration $f^0$ where $t=0$ of each segmentation. To do so, we first calculate the necessary displacements of the centroid of the vessel to the origin of the coordinate system as well as the necessary rotation angles around the *y*- and *z*-axis, so that the centerline of the vessel is parallel to the *x*-axis (here the *x*-axis corresponds to the longitudinal direction of the blood vessel and the centerline is a straight connection between the two endpoints of the vessel). Once this has been done for all segmentations, an averaged translational displacement is calculated from this, as well as the averaged rotation angles about the *y* and *z* axes. The resulting translational and rotational transformation is then equally applied to all segmentations. This guarantees that there is no incorrect overlapping of the segmentations. For later use the *x*-range is determined, which is given by a minimum and a maximum *x*-coordinate between which data points from all segmentations are available. We now consider segmentation ${\varvec{M}}_1$ and transform its Cartesian coordinates into cylindrical coordinates which results in an unwinding of the blood vessel. To ensure a smooth interpolation the unwinding itself is appended to both edges. For each height, first a spline interpolation is performed in the $\phi$-*R* plane (new radial component) and then a spline interpolation in the $\phi$-*x* plane (new *x* component). While the interpolation is also done for the appended unwindings, the interpolation function is evaluated in each case at 36 equally distributed points only in the limits of -$\pi$:$\pi$ (here for orientation to the original 36 degrees of the ultrasonic mesh). Now interpolation is performed along the *x*-axis for each degree position. From the minimum and maximum *x*-coordinates determined before, the equally distributed new *x*-coordinates must be determined, at which the interpolation function is evaluated. Again for orientation to the original mesh, 36 new *x*-positions were determined. After these interpolations have been done for each segmentation, all segmentations show a homogeneous mesh with identical $\phi$- and *x*-coordinates, but different radial coordinates. Last, these radial components of each segmentation are averaged for each point, resulting in a homogeneous mesh for the reference configurations $f^0$ of the segmentations ${\varvec{M}}_1^0$ and ${\varvec{M}}_2^0$ as shown in Fig. 3b.

*Step 2: Assignment of the homogeneous nodes in the reference configuration* $f^0$

So far, only the averaged reference configuration $f^0$ has been created. However, the displacement fields for the current configurations $f^t$ differ for each segmentation, so the newly created homogeneous points of the reference configurations $f^0$ of all segmentations must be tracked over time. In order to meaningfully track the newly created nodes of the homogeneous mesh over one heart cycle, each node must be assigned to a native element of the corresponding segmentation. We first consider only segmentation ${\varvec{M}}_1$ again. For each point of the homogeneous mesh, we compute the Euclidean distance to the four nodes of all native elements on the segmentation. The native element with the smallest Euclidean distance of the nodes is assigned to this point (Fig. 3c). This also has to be done for each native mesh of every segmentation.

*Step 3: Relative position of the homogeneous points in the assigned element of the reference configuration* $f^0$

Now we need to calculate the relative location of each homogeneous point within the assigned native element of the corresponding segmentation for the reference configuration $f^0$. The basic idea is to keep the relative position of this homogeneous point within the native element. So if the native element deforms in the current configurations, the resulting position of the homogeneous point in the new, deformed element is calculated based on the relative position of the reference configuration $f^0$. This can be done by using the isoparametric finite element formulation, which basic procedure is to describe element coordinates and displacements in the form of interpolations using the natural coordinate system of the element. Considering an arbitrary two-dimensional element, the coordinate interpolations are, according to Bathe (2014)

11 $$\begin{aligned} x_P = \sum _{i=1}^{q}h_i x_i \quad ; \quad y_P = \sum _{i=1}^{q}h_i y_i \end{aligned}$$

where $x_P$ and $y_P$ are the local coordinates of an point *P* inside the element and $x_i$, $y_i$, $i = 1, \ldots , q$, are the coordinates of the *q* element nodes $N_1^0$-$N_4^0$. The interpolation functions $h_i$ are defined in the natural coordinate system of the element, which has coordinates *r* and *s* that each vary from $-1:+1$. The fundamental property of interpolation function $h_i$ is that its value in the natural coordinate system is unity at node *i* and zero at all other nodes (Bathe 2014). More specifically, the interpolation functions for a 2D element with four nodes can, again according to Bathe (2014), be expressed in the form

12 $$\begin{aligned} h_1&= \dfrac{1}{4}(1+r)(1+s) \quad ; \quad h_2&= \dfrac{1}{4}(1-r)(1+s)\nonumber \\ h_3&= \dfrac{1}{4}(1-r)(1-s) \quad ; \quad h_4&= \dfrac{1}{4}(1+r)(1-s). \end{aligned}$$

Using Eqs. (11) and (12) we can calculate the coordinates of any point inside the element. If we transform the element into the plane where a local coordinate system is located in the centroid of this element and then further relate Eqs. (11) and (12) to our problem, we declare that $x_{P}$ and $y_{P}$ are the now local coordinates of an assigned homogeneous point *H* as a function of *r* and *s*, and $x_{i}$ and $y_{i}$ are the also now local coordinates of the four element nodes, we get the formulations

13 $$\begin{aligned} x_{H, l}^0 ={}&\dfrac{1}{4} (1+r)(1+s)x_{1, l}^0 + \dfrac{1}{4} (1-r)(1+s)x_{2, l}^0 \nonumber \\&\quad + \dfrac{1}{4} (1-r)(1-s)x_{3, l}^0 \nonumber \\&\quad + \dfrac{1}{4}(1+r)(1-s)x_{4, l}^0 \nonumber \\ y_{H, l}^0 ={}&\dfrac{1}{4} (1+r)(1+s)y_{1, l}^0 + \dfrac{1}{4} (1-r)(1+s)y_{2, l}^0 \nonumber \\&\quad + \dfrac{1}{4} (1-r)(1-s)y_{3, l}^0 \nonumber \\&\quad + \dfrac{1}{4}(1+r)(1-s)y_{4, l}^0 \end{aligned}$$

where $x_{1, l}^0$-$x_{4, l}^0$ and $y_{1, l}^0$-$y_{4, l}^0$ are the positions of the element nodes in local coordinates, $x_{H, l}^0$-$y_{H, l}^0$ is the position of the homogeneous point $H^0$ in local coordinates, and *r*-*s* are the isoparametric parameters of the homogeneous point $H^0$ describing its relative position within the element for the reference configuration, respectively (Fig. 3d). The parameters *r*-*s* are unknown and may vary for every element. Though the calculation of *r*-*s* must then be done iteratively but can, by a simple subtraction, be put into the form

14 $$\begin{aligned} 0={}&\dfrac{1}{4} (1+r)(1+s)x_{1, l}^0 + \dfrac{1}{4} (1-r)(1+s)x_{2, l}^0 \nonumber \\&\quad + \dfrac{1}{4} (1-r)(1-s)x_{3, l}^0 \nonumber \\&\quad + \dfrac{1}{4}(1+r)(1-s)x_{4, l}^0 - x_{H, l}^0 \nonumber \\ 0={}&\dfrac{1}{4} (1+r)(1+s)y_{1, l}^0 + \dfrac{1}{4} (1-r)(1+s)y_{2, l}^0 \nonumber \\&\quad + \dfrac{1}{4} (1-r)(1-s)y_{3, l}^0 \nonumber \\&\quad + \dfrac{1}{4}(1+r)(1-s)y_{4, l}^0 - y_{H, l}^0 \end{aligned}$$

and thereby a minimization problem with the constraint that the *r*-*s* coordinates must run in the bounds $-1:+1$ occurs. To solve Eq. (14) for *r* and *s*, we use the nonlinear least-squares fitting function lsqnonlin which is implemented in MATLAB R2021b (The MathWorks, Inc., Massachusetts, USA) and accepts parameter bounds. Initial values for the algorithm are 0 for both coordinates. These *r*-*s* coordinates will remain stable for the following configurations $f^t$. We assume that the radial position of the homogeneous point in the *z*-axis does not change during all operations. This determination of the *r*-*s* coordinates must be carried out for each homogeneous point of the reference configurations in both segmentations ${\varvec{M}}_1^0$ and ${\varvec{M}}_2^0$.

*Step 4: Tracking the homogeneous points of the reference configuration* $f^0$ *over one heart cycle*

Since the assignment of homogeneous point and native element does not change, the *r*-*s* coordinates found in the reference configuration $f^0$ for a homogeneous point $H^0$ can be used to calculate the new positions $H^t$ in every following current configuration $f^t$ using Eq. (13) by inserting the *x*- and *y*-coordinates of the assigned native element with nodes $N_1^t$-$N_4^t$ of the respective current configuration $x_{1, l}^t$-$x_{4, l}^t$ and $y_{1, l}^t$-$y_{4, l}^t$. Movement of this homogeneous point $H^t$ is than always based on the deformation of the assigned native element measured by ultrasound, what is exactly what we want (Fig. 3e).

*Step 5: Averaging of the newly created homogeneous points*

After steps *2* to *4* have been performed for all segmentations, the resulting new positions $H^t$ have to be averaged. A newly created point $H^0$ of the reference configuration $f^0$ has identical coordinates in all segmentations. However, since each of these points moves individually over time based on the respective segmentation, the tracked points $H_1^t$ and $H_2^t$ may have slightly different coordinates. To ultimately obtain an averaged model, the spatial coordinates of the tracked points $H^t$ of each segmentation are averaged in the last step. This results in an averaged point motion based on two segmentations measured by ultrasound (Fig. 3f). Like initially mentioned, this technique can be applied to any number of segmentations with different number of nodes.

Figure. 6 shows in detail the results for the averaged model at three different locations along the longitudinal axis of the aneurysm, while Fig. 3 gives an overall view of the individual steps for creation as well as showing the averaged model of the entire aneurysm.

## Appendix B: Mathematical formulation of Biot’s in-plane strain tensor

In order to calculate local 2D element strains, the centroid of each element (origin of the local coordinate system) is first dragged to the origin of the global coordinate system with base ($0\mid 0\mid 0$) and then rotated so that it lies in the global *x*-*y* plane. Following Ogden (1997), the resulting nodal coordinates ${\varvec{N}}_{P_k}$, $k = 1,..., 4$ of the element can be calculated using

15 $$\begin{aligned} \begin{aligned} {\varvec{N}}_{P_k} =&{\varvec{R}}_{tot}^{0^{-1}} \cdot {\varvec{N}}_{P{_k}, loc} \\ =&\begin{bmatrix} \hat{{\varvec{e}}}_1{\varvec{e}}_1 &{} \hat{{\varvec{e}}}_1{\varvec{e}}_2 &{} \hat{{\varvec{e}}}_1{\varvec{e}}_3 \\ \hat{{\varvec{e}}}_2{\varvec{e}}_1 &{} \hat{{\varvec{e}}}_2{\varvec{e}}_2 &{} \hat{{\varvec{e}}}_3{\varvec{e}}_3 \\ \hat{{\varvec{e}}}_3{\varvec{e}}_1 &{} \hat{{\varvec{e}}}_3{\varvec{e}}_2 &{} \hat{{\varvec{e}}}_3{\varvec{e}}_3 \end{bmatrix}^{-1} \cdot {\varvec{N}}_{P{_k}, loc}, k = 1,...,4 \end{aligned} \end{aligned}$$

since the scalar product of normalized vectors provides the cosine of the spatial angles and thus describes the required rotations. Here $\hat{{\varvec{e}}}_i$, $i = 1, 2, 3$ are the local basis unit vectors in the element centroid, ${\varvec{e}}_i$, $i = 1, 2, 3$ are the global basis unit vectors for basis ($0\mid 0\mid 0$), ${\varvec{R}}_{tot}^{0^{-1}}$ is the transformation matrix and ${\varvec{N}}_{P{_k}, loc}$ is the connection vector between element centroid and the respective element node in local coordinates. The local 1–2 plane is thus transformed into the global *x*-*y* plane, and the 3- or *z*-components disappear. For the linkage of the geometry of a 4-node element lying in the physical 2D space with that in the isoparametric space, the interpolation functions according to Eq. (12) are also used here. The elements lying in the physical space are transformed into the isoparametric space by means of a Jacobi transformation. The Jacobi matrix acts as a “deformation gradient” of an arbitrarily shaped element into an isoparametric element. However, since the interpolation functions must be derived according to the coordinates in physical space and not according to the isoparametric parameters *r* and *s*, the Jacobi matrix must be shaped like

16 $$\begin{aligned} \begin{aligned} \left[ \begin{array}{ll} \frac{\partial f(r,s)}{\partial x^0} \\ \frac{\partial f(r,s)}{\partial y^0} \end{array}\right] =&\frac{1}{D} \left[ \begin{array}{ll}\quad \frac{\partial y^0}{\partial s} &{} -\frac{\partial y^0}{\partial r} \\ -\frac{\partial x^0}{\partial s} &{} \quad \frac{\partial x^0}{\partial r} \end{array}\right] \left[ \begin{array}{ll}\frac{\partial x^t(r,s)}{\partial r} \\ \frac{\partial y^t(r,s)}{\partial s} \end{array}\right] \\ \text {where} \quad \frac{1}{D} =&\frac{1}{ \frac{\partial x^0}{\partial r} \frac{\partial y^0}{\partial s} - \frac{\partial x^0}{\partial s} \frac{\partial y^0}{\partial r}} \end{aligned} \end{aligned}$$

according to Bathe (2014). Here $x^0$ and $y^0$ are the coordinate interpolation functions according to Eq. (11) in the reference configuration $f^0$ and $x^t$ and $y^t$ correspond to Eq. (11) in the following current configurations $f^t$. The expression ${\frac{\partial x^0}{\partial r} \frac{\partial y^0}{\partial s} - \frac{\partial x^0}{\partial s} \frac{\partial y^0}{\partial r}}$ is the determinant of the Jacobian matrix. By using the Lagrangian formulation, these need to be calculated only once for each element of the reference configuration. The resulting distortions of a material point between two time steps from the reference configuration $f^0$ to the current configuration $f^t$ is described by means of the deformation gradient ${\varvec{F}}_0^t$. For transformation into an isoparametric element, it is derived from the derivatives of the coordinate interpolations of the current configuration $x^t$ and $y^t$ to the coordinates of the reference configuration $x^0$ and $y^0$ by (Bathe 2014)

17 $$\begin{aligned} {\varvec{F}}_0^{t} = \left[ \begin{array}{ll} \frac{\partial x^t}{\partial x^0} &{} \frac{\partial x^t}{\partial y^0} \\ \frac{\partial y^t}{\partial x^0} &{} \frac{\partial y^t}{\partial y^0} \end{array}\right] \quad . \end{aligned}$$

Since the four entries of the deformation gradient are also derivatives of the interpolation functions according to coordinates of the physical space, the same prescription as for Eq. (16) must be used here in each case (Bathe 2014). The deformation gradient ${\varvec{F}}_0^{t}$ is not objective due to its contained rotation and needs a left polar decomposition by means of

18 $$\begin{aligned} {\varvec{F}}_0^{t} = {\varvec{R}}_0^t {\varvec{U}}_0^t \end{aligned}$$

into the (unknown) rotation part ${\varvec{R}}_0^t$ and the stretches ${\varvec{U}}_0^t$. The right Cauchy–Green distortion tensor corresponds by

19 $$\begin{aligned} {\varvec{C}}_0^t = {\varvec{F}}_0^{t^T} {\varvec{F}}_0^{t} = {{\varvec{U}}_0^t}^2 \end{aligned}$$

to the square of the stretches contained in the polar decomposition (18) and must, because of the existing values on the minor diagonal, first be transformed into the principal axis system via the solution of the eigenvalue problem

20 $$\begin{aligned} {\varvec{C}}_0^t {\varvec{p}}_0^t = \varvec{\lambda } {\varvec{p}}_0^t \quad . \end{aligned}$$

Here we obtain the right Cauchy–Green distortion tensor ${\varvec{C}}_{p_0}^{t}$ and the matrix of eigendirections ${\varvec{p}}_0^t$ in the principal axis system. On the diagonal of ${\varvec{C}}_0^t {\varvec{p}}_0^t$ the squared principal stretches $\lambda _1^2$ and $\lambda _2^2$ are included as calculated eigenvalues. The secondary diagonals are equal to zero here, so the Biot’s strain tensor in the principal axis system can be calculated with Eq. (19) and subtraction of the unit tensor ${\varvec{I}}$ to the form

21 $$\begin{aligned} \begin{aligned} \varvec{\epsilon }_{{\varvec{p}}_0}^{t} =&{\varvec{U}}_{{\varvec{p}}_0}^{t} - {\varvec{I}} = \sqrt{{\varvec{C}}_{{\varvec{p}}_0}^{t}} - {\varvec{I}} \\ =&\begin{bmatrix} \lambda _{P_1} &{} 0 \\ 0 &{} \lambda _{P_2} \end{bmatrix} - {\varvec{I}} = \begin{bmatrix} \epsilon _{P_1} &{} 0 \\ 0 &{} \epsilon _{P_2} \end{bmatrix} \quad , \end{aligned} \end{aligned}$$

where $\epsilon _{P_1}$ and $\epsilon _{P_2}$ are the principle strains (Bathe 2014). To find our way back to the global system, a 2D total transformation matrix can be formed similar to ${\varvec{R}}_{tot}^{0^{-1}}$ in Eq. (15), whose components are calculated via the scalar product of the eigenvectors $\hat{{\varvec{e}}}_{1,p}$ and $\hat{{\varvec{e}}}_{2,p}$ of the right Cauchy–Green distortion tensor and the unit vectors ${\varvec{e}}_1$ and ${\varvec{e}}_2$ of the global system (Ogden 1997). Finally, the Biot’s strain tensor in the global system can then be described by

22 $$\begin{aligned} \begin{aligned} \varvec{\epsilon }_0^{t} =&{\varvec{R}}_{tot}^{0} \varvec{\epsilon }_{{\varvec{p}}_0}^{t} {\varvec{R}}_{tot}^{0^{T}} \\ =&\begin{bmatrix} \hat{{\varvec{e}}}_{1,p}{\varvec{e}}_1 &{} \hat{{\varvec{e}}}_{1,p}{\varvec{e}}_2 \\ \hat{{\varvec{e}}}_{2,p}{\varvec{e}}_1 &{} \hat{{\varvec{e}}}_{2,p}{\varvec{e}}_2 \end{bmatrix} \begin{bmatrix} \epsilon _{P_1} &{} 0 \\ 0 &{} \epsilon _{P_2} \end{bmatrix} \begin{bmatrix} \hat{{\varvec{e}}}_{1,p}{\varvec{e}}_1 &{} \hat{{\varvec{e}}}_{1,p}{\varvec{e}}_2 \\ \hat{{\varvec{e}}}_{2,p}{\varvec{e}}_1 &{} \hat{{\varvec{e}}}_{2,p}{\varvec{e}}_2 \end{bmatrix}^T \\ =&\begin{bmatrix} \epsilon _{11} &{} \frac{1}{2}\epsilon _{12} \\ \frac{1}{2}\epsilon _{12} &{} \epsilon _{22} \end{bmatrix} \quad . \end{aligned} \end{aligned}$$

Here $\epsilon _{11}$, $\epsilon _{22}$ and $\epsilon _{12}$ are the strains in longitudinal and circumferential direction and in-plane shear, respectively.
